# Epidemiology of hepatitis B, C and D in Malawi: systematic review

**DOI:** 10.1186/s12879-018-3428-7

**Published:** 2018-10-12

**Authors:** Alexander J Stockdale, Collins Mitambo, Dean Everett, Anna Maria Geretti, Melita A Gordon

**Affiliations:** 1 Malawi Liverpool Wellcome Trust Clinical Research Programme, Chichiri 3, PO Box 30096, Blantyre, Malawi; 2 Institute of Infection and Global Health, University of Liverpool, Ronald Ross Building, 8 West Derby Street, Liverpool, L69 7BE UK; 3 HIV and AIDS Department, Malawi Ministry of Health, PO Box 30377, Lilongwe, Malawi; 4 MRC Centre for Inflammation Research, The Queen’s Medical Research Institute, University of Edinburgh, 47 Little France Crescent, Edinburgh, EH16 4TJ UK

**Keywords:** Epidemiology, Viral hepatitis, Hepatitis B, Hepatitis C, Hepatitis D, Malawi, Sub-Saharan Africa

## Abstract

**Background:**

Viral hepatitis is an important public health issue in sub-Saharan Africa. Due to rising mortality from cirrhosis and hepatocellular carcinoma and limited implementation of screening and treatment programmes, it has been characterised as a neglected tropical disease. Synthesis of the existing evidence on the epidemiology of viral hepatitis B, C and D in Malawi is required to inform policy and identify research gaps.

**Methods:**

We searched Pubmed, EMBASE and Scopus for studies reporting the epidemiology of viral hepatitis B, C and D in Malawi from 1990 to 2018. Articles reporting prevalence estimates were included provided they described details of participant selection, inclusion criteria and laboratory methods (detection of HBsAg, anti-HCV or anti-HDV antibody, HCV antigen or HCV RNA or HDV RNA). We assessed study quality using a prevalence assessment tool. Where appropriate, a pooled prevalence was calculated using a DerSimonian Laird random effects model.

**Results:**

Searches identified 199 studies, 95 full text articles were reviewed and 19 articles were included. Hepatitis B surface antigen (HBsAg) seroprevalence was assessed in 14 general population cohorts. The pooled prevalence among adults was 8.1% (95% CI 6.1, 10.3). In 3 studies where HBsAg was stratified by HIV status, no effect of HIV on HBsAg prevalence was observed (OR 1.2 (95% CI: 0.8, 1.6, *p* = 0.80)). In a single study of HIV/HBV infected individuals, anti-hepatitis D antibody (anti-HDV) prevalence was low (1.5%). HCV antibody prevalence (anti-HCV) ranged from 0.7 to 18.0% among 12 cohorts in general populations. Among three studies which used PCR to confirm current infection, the pooled rate of HCV RNA confirmation among anti-HCV positive individuals was only 7.3% (95% CI: 0.0, 24.3).

**Conclusions:**

Hepatitis B is highly prevalent in Malawi. There is a paucity of epidemiological data from rural areas where 85% of the population reside, and the Northern region. Priority research needs include large-scale representative community studies of HBV, HDV and HCV seroprevalence, assessment of children following introduction of the HBV vaccine in 2002, prevalence estimates of viral hepatitis among individuals with cirrhosis and HCC and data on HCV prevalence using PCR confirmation, to support a viral hepatitis strategy for Malawi.

**Electronic supplementary material:**

The online version of this article (10.1186/s12879-018-3428-7) contains supplementary material, which is available to authorized users.

## Background

Viral hepatitis is the principal cause of liver cirrhosis and hepatocellular carcinoma (HCC) in sub-Saharan Africa [1]. Due to limited availability of screening and treatment programmes, it has been characterised as a neglected tropical disease [2]. In contrast with HIV, malaria and tuberculosis, where public health interventions have resulted in substantial reductions in mortality, viral hepatitis-associated mortality is rising: cirrhosis and HCC were the cause of an estimated 3.2% of adult deaths in 2005, rising to 4% in 2016 [3, 4]. In Malawi, the cirrhosis-associated mortality rate has been ranked in the top global decile [5]. Across Southern Africa, an estimated 50–64% of cases of HCC are attributable to viral hepatitis, and with limited treatment options outcomes are poor with an estimated annual mortality to incidence ratio of 96% [6–8]. HCC has been shown to occur in a younger age group among individuals in sub-Saharan Africa and in HBV-associated cases (relative to HCV-associated cases), contributing to increased disease impact [9, 10].

Data on the epidemiology of viral hepatitis are required to inform an effective public health response. In the Global Health Sector Strategy on Viral Hepatitis 2016–2021, the World Health Organisation (WHO) has identified the need to define the national disease burden and strategically target limited resources to counter the local epidemic. There is a WHO call for data on transmission and risk factors, to identify specific populations at risk and to quantify the health burden in terms of cirrhosis and hepatocellular carcinoma [11].

The Malawi Ministry of Health (MoH) has resolved to respond to viral hepatitis in a concerted and strategic manner. As part of the response, a National Viral Hepatitis Unit has been created in the MoH to guide the direction of policy and practice. In order to consolidate the current available evidence on epidemiology of viral hepatitis, identify the gaps in knowledge, practice and policy, we aimed to conduct a systematic review of all published epidemiological data on the prevalence of chronic hepatitis B, C and D in Malawi and identify further research needs.

## Methods

**Fig. 1:**
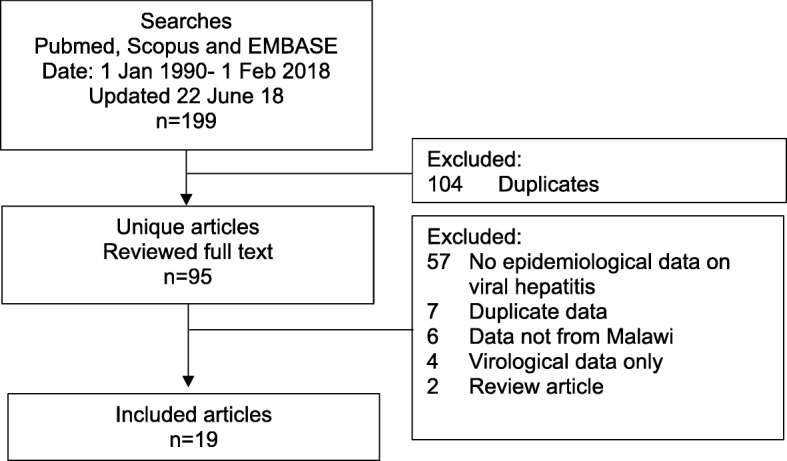
Flowchart of literature searches

Searches were performed in Pubmed, Scopus and EMBASE using the search terms Malawi AND (hepatitis or hepatitis B or HBV or HBsAg or hepatitis C or HCV or anti-HCV or HCV antibody or core HCV antigen or HCVcAg or HCV RNA or hepatitis D or HDV or anti-HD or anti-HDV or HDV IgG or HDV RNA or viral hepatitis). (Additional file 1: Table S1) Medical subject headings [MeSH] in Pubmed and EMBASE thesaurus tools were employed. Searches were restricted to publications between 1 Jan 1990 and 1 February 2018 with a search update on 22 June 18, to identify published data from the past 28 years (Fig. 1).

Data were grouped into two categories: “general populations” which provided data from potentially representative community samples, pregnant women, or blood donors; “HIV positive populations” adults or women, or children receiving routine HIV care, and “special groups”, comprising populations likely to be unrepresentative of the general population such as medical inpatients, prisoners or medical students.

Studies reporting detection of hepatitis B surface antigen (HBsAg), or total or IgG anti-hepatitis delta antibody (anti-HDV) or HDV RNA among HBsAg positive people, or anti-hepatitis C antibody (anti-HCV), hepatitis C core antigen (HCVcAg) or HCV RNA were included, provided they presented details of selection and inclusion criteria and described the laboratory methods used.

### Data extraction and quality assessment

We conducted this review in accordance with PRISMA guidelines [12]. We extracted details of study design, participant characteristics (age and gender distribution, population group), sampling method, dates, study locations, laboratory test used and prevalence estimates. A quality assessment tool for prevalence estimates was used and study quality was independently evaluated by two authors (AS, CM) with discordance resolved by discussion [13].

### Statistical analysis

Confidence intervals for prevalence were calculated using the Wilson method. Pooled seroprevalence for hepatitis B was calculated with the DerSimonian-Laird random-effects model with Freeman-Tukey double arcsine transformation [14, 15]. A random effects model was applied due to anticipated heterogeneity. Study heterogeneity was assessed using the I^2^ statistic. Analyses were performed in Stata release 14.2 (College Station, TX, USA) using the metaprop package [16].

## Results

The literature search identified 199 studies. Following removal of duplicates, 95 full-text articles were reviewed and 19 studies that reported epidemiological data on hepatitis B, C and D in diverse populations in Malawi were included (Fig. 1).

### Description of included studies

**Fig. 2:**
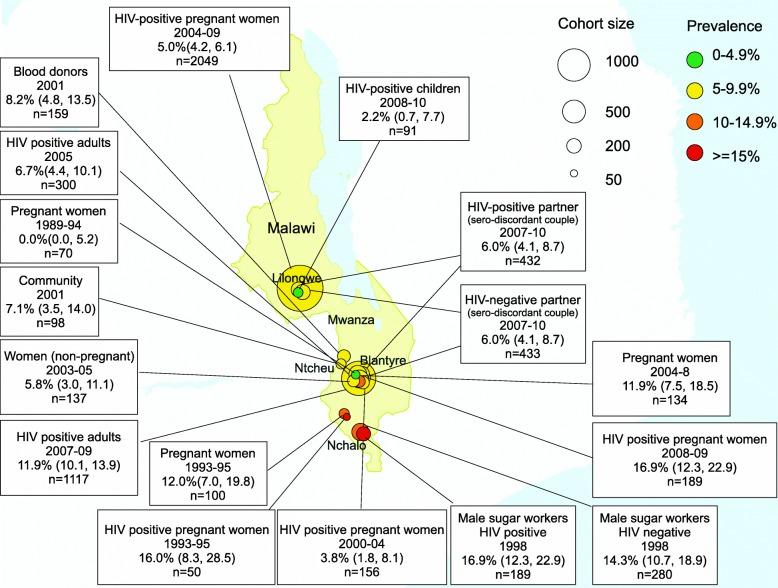
HBsAg seroprevalence in Malawi, published data 1990–2018

The 19 included studies described a total of HBsAg seroprevalence data from 16 different cohorts that were general or HIV-positive populations (Table 1; Fig. 2) and three cohorts from specific unrepresentative subgroups (Table 2); hepatitis D antibody (anti-HDV) data was available from a single study (Table 3) and hepatitis C antibody (anti-HCV) data was available from 15 general or HIV-positive cohorts (Table 4; Fig. 4) and from four cohorts describing specific subgroups (Table 5). Fourteen of 18 studies were from urban centres.

**Table 1:** Hepatitis B surface antigen (HBsAg) seroprevalence in Malawi: published data from 1990 to 2018

Population	Ref	Year	Location	Laboratory method	Prevalence (n/total)	Prevalence (%), (95% CI)
General Populations						
Pregnant women	[20]	1989–1994	QECH, Blantyre	MONOLISA HBsAg ULTRA (Biorad)	0/70	0.0 (0.0, 5.2)
Pregnant women, at delivery	[18]	1993–1995	Shire Valley	Bioelisa HBsAg (Biokit, S.A.)	12/100	12.0 (7.0, 19.8)
Pregnant women	[20]	2004–2008	QECH, Health Centres Blantyre	MONOLISA HBsAg ULTRA (Biorad)	16/134	11.9 (7.5, 18.5)
Male workers at sugar estate	[17]	1998	Nchalo	Auszyme monoclonal EIA (Abbott)	40/280	14.3 (10.7, 18.9)
Community, rural adults	[20]	2001	Mwanza District	MONOLISA HBsAg ULTRA (Biorad)	7/98	7.1 (3.5, 14.0)
Non-pregnant women (intravaginal MTZ gel RCT)	[20]	2003–2005	QECH, Blantyre	MONOLISA HBsAg ULTRA (Biorad)	8/137	5.8 (3.0, 11.1)
HIV-negative partners in a serodiscordant couple	[19]	2007–2010	Blantyre Lilongwe	HBsAg ELISA NS	26/433	6.0 (4.1, 8.7)
Blood donors	[21]	2001	Ntechu	HBsAg ELISA NS	13/159	8.2 (4.8, 13.5)
HIV-positive populations						
HIV-positive pregnant women, at delivery	[18]	1993–1995	Shire Valley	Bioelisa HBsAg (Biokit, S.A.)	8/50	16.0 (8.3, 28.5)
HIV-positive pregnant women	[20]	2000–2004	QECH, Blantyre	MONOLISA HBsAg ULTRA (Biorad)	6/156	3.8 (1.8, 8.1)
HIV-positive pregnant women	[22]	2004–2009	Lilongwe	Vitros Chemiluminescence Immunoassay (Ortho Clinical Diagnostics)	103/2049	5.0 (4.2, 6.1)
HIV-positive pregnant women	[23]	2008–2009	Blantyre	Murex HBsAg Version 3 with Confirmatory Assay (Murex Biotech)	27/309	8.7 (6.1, 12.4)
HIV positive: male workers at sugar estate	[17]	1998	Nchalo	Auszyme monoclonal EIA (Abbott)	32/189	16.9 (12.3, 22.9)
HIV-positive adults	[24]	2005	QECH, Blantyre	Bioelisa HBsAg (Biokit, S.A.)	20/300	6.7 (4.4, 10.1)
HIV positive adults, ART starters	[25]	2007–2009	QECH, Blantyre	Bioelisa HBsAg (Biokit, S.A.)	133/1117	11.9 (10.1, 13.9)
HIV-positive adults in sero-discordant couple	[19]	2007–2010	Blantyre Lilongwe	HBsAg ELISA NS	26/432	6.0 (4.1, 8.7)
HIV-infected children	[26]	2008–2010	Lilongwe	Genetic Systems HBsAg 3.0 (Bio-Rad)	2/91	2.2 (0.6, 7.7)

*Abbreviations*: *QECH* Queen Elizabeth Central Hospital, Blantyre. This is a tertiary referral hospital, *MTZ* metronidazole, *RCT* randomised controlled trial. Biorad: HBsAg ELISA, Biorad, Hercules, CA, USA; Bioelisa: HBsAg 3.0 Biokit SA Barcelona, Spain; Ortho Clinical Diagnositics: Raritan, New Jersey, United States: Siemens: ADVIA Centaur, Siemens, Munich, Germany; Abbott: Murex HBsAg, Abbott, Illinois, USA; HBsAg ELISA NS- manufacturer not specified

**Table 2:** HBsAg seroprevalence among special unrepresentative populations in Malawi: Published data from 1990 to 2018

Population	Ref	Year	Location	Laboratory method	Prevalence (n/total)	Prevalence (%, (95% CI))
Adult medical inpatients	[27]	2004	Medical ward, QECH, Blantyre	Determine HBsAg Rapid Test (Alere)	34/194	17.5 (12.8, 23.5)
Prisoners	[28]	2007	Chichiri Prison, Blantyre	HBsAg kit (Abbott)	5/164	3.0 (1.3, 6.9)
Medical students	[29]	2013	College of Medicine, Blantyre	SD Bioline Rapid Test (Alere)	0/89	0.0 (0.0, 4.9)

*Abbreviations*: *QECH* Queen Elizabeth Central Hospital, *ART* antiretroviral therapy

**Table 3:** Published data on hepatitis D seroprevalence in Malawi among HBsAg positive individuals

Population	Ref	Year	Location	Method	Prevalence (n/total)	Prevalence (%, (95% CI))
HIV-HBV infected adults	[30]	2007–2009	HIV clinic, QECH Blantyre	1. ETI-AB-DELTAK (Diasorin)	2/133	1.5 (0.4, 5.3)
				2. HDV RNA PCR (in-house)	0/133	0.0 (0.0, 2.8)

**Table 4:** Published data on hepatitis C seroprevalence in Malawi

Population	Ref	Year	Location	Method	Prevalence (n/total)	Prevalence (%, (95% CI))
General Populations						
Pregnant women	[20]	1989–1994	QECH, Blantyre	Anti-HCV (Biorad)	2/70	2.9 (0.8, 9.8)
Pregnant women, at delivery	[18]	1993–1995	Shire Valley	Ortho anti-HCV (Ortho Diagnostics)	18/100	18.0 (11.7, 26.7)
Pregnant women	[20]	2004–2008	QECH, Health Centres Blantyre	Anti-HCV (Biorad)	8/138	5.8 (3.0, 11.0)
Community, rural adults	[20]	2001	Mwanza District	Anti-HCV (Biorad)	9/99	9.0 (4.9, 16.4)
Non-pregnant women (intravaginal MTZ gel RCT)	[20]	2003–2005	QECH, Blantyre	Anti-HCV (Biorad)	9/146	6.1 (3.3, 11.3)
Male workers at sugar estate	[17]	1998	Nchalo	Ortho anti-HCV (Ortho Diagnostics)	35/279	10.0 (7.0, 14.1)
Blood donors	[32]	1996	KCH, Lilongwe	Anti-HCV EIA (Roche) Confirmed with Anti-HCV (Abbott)	4/100	4.0 (1.6, 9.8)
Blood donors	[21]	2001	Ntechu	1. Murex anti-HCV	10/148	6.8 (3.7, 12.0)
				2. HCV RNA by in-house PCR	1/140	0.7 (0.1, 3.9)
HIV positive populations						
HIV-positive pregnant women, at delivery	[18]	1993–1995	Shire Valley	Ortho anti-HCV (Ortho Diagnostics)	6/50	12.0 (5.6, 23.8)
HIV-positive pregnant women	[23]	2008–2009	Blantyre	1. Innotest HCV Ab IV (Innogenetics),	8/309	2.6 (1.3, 5.0)
				2. Versant HCV RNA 1.0 assay (Siemens)	1/309	0.3 (0.1, 1.8)
HIV positive patients	[24]	2005	QECH, Blantyre	Monolisa HCV Ag-Ab (Biorad) confirmed with ADVIA Centaur anti-HCV) and InnoLIA HCV immunoassay (Innogenetics)	17/300	5.7 (3.6, 8.9)
HIV-positive male workers at sugar estate	[17]	1998	Nchalo	Ortho anti-HCV Ab (Ortho Clinical Diagnostics)	28/280	10.0 (7.0, 14.1)
HIV-positive pregnant women	[20]	2000–2004	QECH, Blantyre	Anti-HCV (Biorad)	8/148	5.4 (2.8, 10.3)
HIV positive adults starting ART	[31]	2014–15	Lilongwe	1. HCV IgG Architect (Abbott)	5/227	2.2 (0.9, 5.1)
				2. RealTime HCV RNA (Abbott)	0/227	0.0 (0.0, 1.7)
HIV positive patients on ART for > 10 years	[33]	2014–16	Chiradzulu	OraQuick HCV Rapid antibody test (Orasure)	2/385	0.5 (0.1, 1.9)

*Abbreviations*: *QECH* Queen Elizabeth Central Hospital, *HCV* hepatitis C virus Biorad: Hercules, CA, USA; Ortho Clinical Diagnostics: Raritan, New Jersey, United States; Roche: Basel Switzerland; Abbott: Illinois, USA; Innogenetics: Ghent, Belgium; Siemens: Munich, Germany; Orasure: Bethlehem, Pennsylvania, United States

**Table 5:** Published data on hepatitis C seroprevalence among special unrepresentative populations in Malawi: Published data from 1990 to 2018

Population	Ref	Year	Location	Method	Prevalence (n/total)	Prevalence (%, (95% CI))
Prisoners	[28]	2007	Chichiri Prison, Blantyre	Anti-HCV (Biotec)	0/164	0.0 (0.0, 2.3)
Adult inpatients (Dermatology, Urology)	[32]	1996	KCH, Lilongwe	Anti-HCV EIA (Roche) Confirmed with Anti-HCV (Abbott)	13/333	3.9 (2.3, 6.6)
Adult medical inpatients	[27]	2004	Medical ward, QECH, Blantyre	HCV Ag/Ab (Monolisa, Biorad) confirmed with Immunoassay (Innogenetics)	9/202	4.5 (2.4, 8.2)
Malawian women and children with childhood malignancies	[34]	2006–10	QECH, Blantyre	HBV ELISA (MP Biomedicals)	Mothers: 2/418	0.5 (0.1, 1.7)
				Confirmed by HCV BLOT (MP Biomedicals)	Children: 1/418	0.2 (0.0, 1.3)

*Abbreviations*: Biotec: Dorset, United Kingdom; Roche: Basel, Switzerland; Abbott: Illinois, USA; Innogenetics: Ghent, Belgium; MP Biomedicals: California, USA*KCH* Kamuzu Central Hospital, *QECH* Queen Elizabeth Central Hospital, *HCV* hepatitis C virus

### Hepatitis B prevalence

**Fig. 3:**
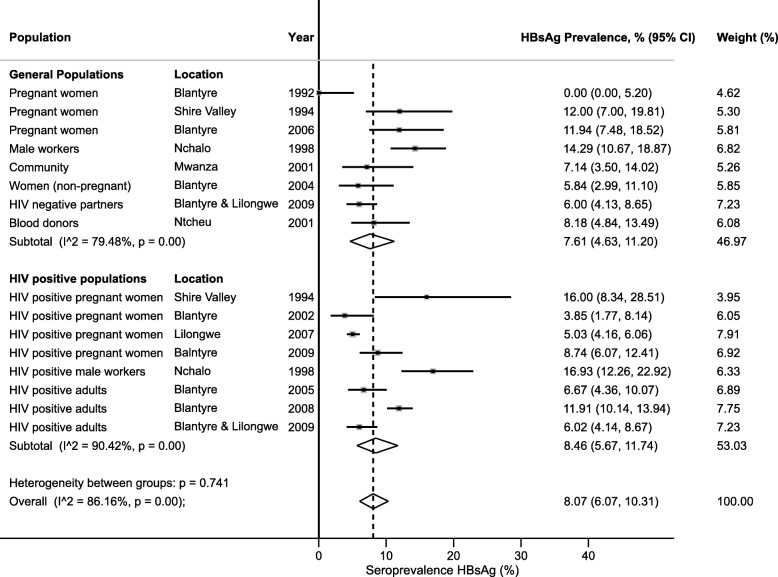
Forest plot of HBsAg prevalence in general and HIV-positive populations, Malawi 1990–2018

HBsAg seroprevalence estimates ranged from 0.0 to 14.3% in general populations and 3.8 to 16.0% in HIV positive populations (Table 1). One small study reporting from HIV positive children aged 3 months - 15 years (median 36 months) reported seroprevalence of 2.2% [95% confidence interval (CI) 0.6, 7.7]. This study did not estimate HBV vaccine efficacy as the vaccine was introduced in Malawi in 2002 and both vaccinated and non-vaccinated cohorts were combined. Pooled estimates of HBsAg seroprevalence among adult general populations was 7.6% (95% CI 4.6, 11.2) and 8.5 (95% CI 5.7, 11.7) in HIV positive populations (Fig. 3). The overall pooled estimate of HBsAg seroprevalence in adults was 8.1% (95% CI 6.1, 10.3).

**Fig. 4:**
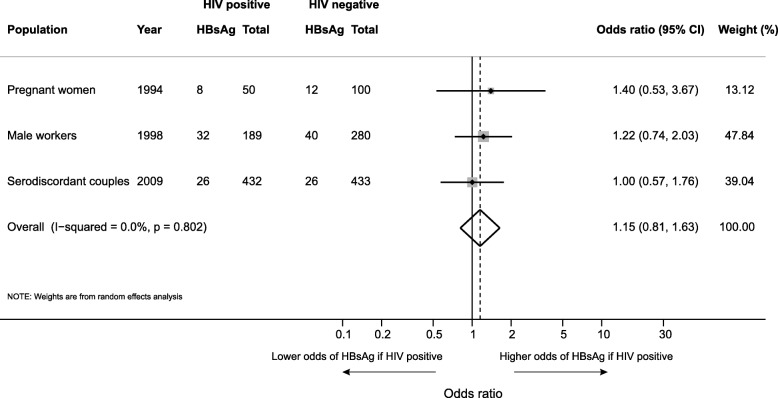
Odds ratio of HBsAg seropositivity according to HIV status

No significant difference in HBsAg prevalence was noted between HIV-positive and -negative populations (*p* = 0.74). The effect of HIV status on HBV seroprevalence was assessed directly in three studies, with a total of 1484 participants, that tested HBsAg prevalence, stratified by HIV status within the same population. These populations comprised male workers at a sugar factory (*n* = 469) [17], pregnant women recruited at delivery (*n* = 150) [18] and HIV positive and negative serodiscordant couples recruited for a randomised control trial of antiretroviral therapy for prevention of transmission (*n* = 865) [19]. Among the three groups, the odds ratio of HBsAg positivity among HIV positive compared to HIV negative individuals from within the same population was 1.2 (95% CI 0.8, 1.6, *p* = 0.80), indicating no evidence of association between HBV infection and HIV infection status. (Fig. 4).

Studies among three unrepresentative groups deemed at altered risk of HBV infection: (adult medical inpatients, prisoners and medical students) found HBsAg prevalence rates of 17.5%, 3.0% and 0% respectively (Table 2).

### Hepatitis D prevalence

A single study was available reporting HDV prevalence among HIV/HBV co-infected individuals commencing ART in Blantyre [30] (Table 3). This demonstrated anti-HDV prevalence of 2/133 (1.5%) but none of the participants were HDV RNA PCR positive.

### Hepatitis C prevalence

**Fig. 5:**
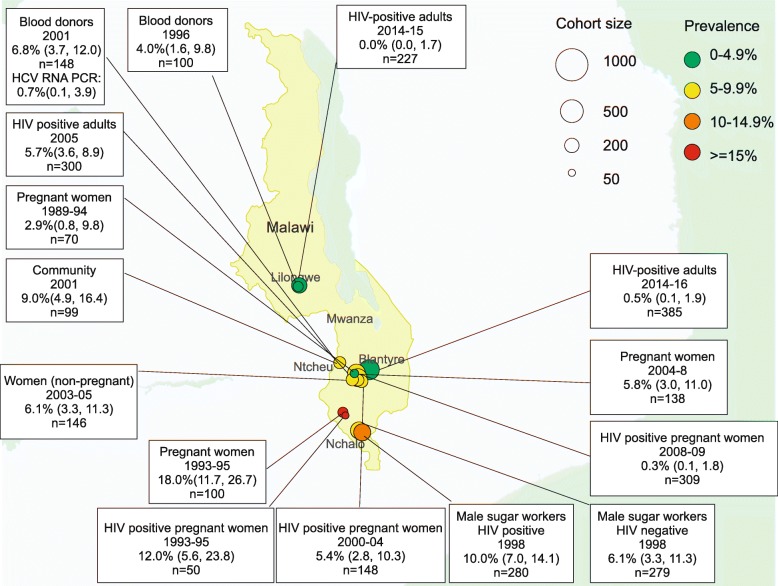
Prevalence of hepatitis C antibody: published data 1990–2018

Among general populations, anti-HCV prevalence ranged from 0.7 to 18.0% and among HIV-positive populations from 0.0 to 12.0%. (Table 4, Fig. 5) Three studies confirmed active HCV infection using RNA PCR. These comprised a study of HIV-positive adults commencing ART in Lilongwe [31], a study of blood donors in Ntcheu [21] and a study of HIV-positive pregnant women in Blantyre [23]. In these studies, anti-HCV prevalence was 2.2, 6.8 and 2.6% respectively but HCV RNA PCR demonstrated active HCV prevalence of 0, 0.7 and 0.3% respectively, with a pooled rate of HCV RNA confirmation among anti-HCV positive participants of 7.3% (95% CI 0.0–24.3).

Among four studies assessing HCV prevalence in unrepresentative special subgroups comprising: prisoners; medical inpatients in Blantyre and Lilongwe; and children with malignancies and their mothers, the prevalence of anti-HCV was 0; 3.9 and 4.5; 0.2 and 0.5% respectively (Table 5).

## Discussion

In this systematic review, we have compiled the existing epidemiological evidence on HBV, HCV and HDV prevalence in Malawi and have highlighted a number of key findings and important knowledge gaps. Data from studies reporting from general and HIV-infected populations showed a pooled HBsAg seroprevalence estimate of 8.1% (95% CI 6.1, 10.3). This finding is in keeping with regional estimates from Mozambique (8.3%), Tanzania (7.2%) and Zambia (6.1%) [35]. Our study has benefitted from the inclusion of significantly more data than previous estimates for Malawi [35, 36]. We noted that available data were biased toward the two main urban centres of Lilongwe and Blantyre, that the Northern region was under-represented and that there were no nationally representative community survey data.

Hepatitis C antibody seroprevalence estimates ranged from 2.9 to 18% from general or HIV-infected populations. Among the three available studies that reported HCV RNA confirmation, only 7.3% of 676 participants with anti-HCV antibody were confirmed to have HCV RNA replication. This finding has been consistent with other cohorts across the region and highlights issues with using anti-HCV as the basis for obtaining epidemiological estimates in the absence of confirmatory testing [37]. Confirmation of anti-HCV results with PCR or core HCV antigen testing are required to obtain reliable prevalence estimates [38]. Accordingly, due to the paucity of studies reporting PCR data, a pooled HCV prevalence estimate was not provided in this review. Furthermore, an assessment of possible association between HCV, HBV and HIV infection was not possible based on the limited data. Based on the available evidence, it is likely that HCV prevalence is low in Malawi, and was below 1% in all studies using RNA confirmation [21, 23, 31], but larger representative samples employing confirmatory PCR testing are required to confirm these findings. Further work to establish whether false positive anti-HCV antibody tests or failure of HCV RNA assays to detect local HCV strains is required, particularly in view of the paucity of available genotypic HCV data from sub-Saharan Africa [39].

Only a single study reporting HDV prevalence was available, demonstrating a low rate of anti-HDV among HIV/HBV co-infected patients in Blantyre (1.5%), with none of the participants showing replication of HDV RNA by PCR. This finding is in keeping with available limited data demonstrating a low rate of HDV seroprevalence from Southern Africa relative to Central or West Africa, though the paucity of available data from the Southern Africa region should be noted [30]. Due to the rapid progression to fatal liver disease associated with HBV/HDV superinfection or co-infection, cross-sectional community estimates of HDV seroprevalence are unlikely to reliably estimate the true burden of disease caused by HDV. Studies of hospitalised patients with well-characterised liver disease are required and will facilitate the ascertainment of the attributable fraction of viral hepatitis to liver disease [40].

There are several limitations in this analysis, highlighted by our assessment of study quality (Additional file 2: Table S2). The epidemiological evidence presented in this study is drawn from predominantly small cohorts studies in diverse populations employing convenience sampling. A striking bias toward urban centres was observed with only four of 18 included studies drawn from rural areas, despite an estimated 85% of the Malawian population residing in rural areas [41]. There were no available data from the Northern region of Malawi, where 13% of the population live [41]. To overcome these issues of lack of nationally representative unbiased community data, the use of the demographic health survey using dried blood spot sampling represents a promising solution. Dried blood spots have excellent diagnostic performance relative to venous blood sampling for HBsAg and anti-HCV screening and this method has been recently recommended for large surveys by the WHO [38]. Use of dried blood spots for hepatitis D screening of the demographic health survey has recently been used in Burkina Faso [42], and represent an efficient method to obtain samples without requiring a cold chain or venepuncture.

The finding of lack of an association between hepatitis B seroprevalence and HIV status is in keeping with previous studies from sub-Saharan Africa [43]. This is likely due to distinct transmission epidemiology, with hepatitis B predominantly acquired perinatally or horizontally in early childhood, and HIV acquired predominantly during adolescence or adulthood by sexual transmission in sub-Saharan Africa. By contrast, recent evidence of incident transmission of HBV in HIV-infected adults has highlighted the risk of HBV acquisition in adulthood [44]. Hepatitis B vaccination is provided as a component of the pentavalent vaccine (also containing, diphtheria, tetanus, pertussis and *Haemophilis influenzae* type B) in the expanded programme of immunisation schedule for Malawian infants, provided at 6, 10 and 14 weeks since 2002. The Demographic Health Survey 2015–16 estimated 3-dose coverage of the vaccine of 93.0%, with consistently high coverage exceeding 90%, regardless of socioeconomic status or geographic location [45]. The WHO has recently proposed that gathering data on hepatitis B seroprevalence among a vaccinated cohort at 5 years of age is a priority in order to generate evidence on the efficacy of HBV vaccination programmes and this is a priority area for research highlighted by this review [46].

## Conclusions

Hepatitis B is highly prevalent in Malawi with an estimated seroprevalence among the general population of 8.1%. HCV prevalence was below 1% in three general population cohorts that used nucleic amplification confirmatory testing. There is a need for representative unbiased community seroprevalence estimates of HBV, HDV and HCV prevalence. These should include confirmatory PCR testing to establish reliable HCV prevalence estimates. Future studies examining seroprevalence among community samples, with a particular focus on rural areas and the Northern region, are required. Assessment of the effectiveness of the hepatitis B vaccination programme introduced in 2002 and data on HDV prevalence among HBsAg positive individuals represent further research priorities. Prevalence estimates of viral hepatitis among people with well-characterised liver disease with cirrhosis and HCC are required to ascertain the attributable fraction and burden of disease. These data will help to support a viral hepatitis strategy for Malawi, facilitate the introduction of screening and treatment programmes for HBV and HCV and begin to reverse the current trend of increasing viral hepatitis-associated mortality.

## Additional files

Additional file 1: **Table S1.** Assessment of quality of included studies, Assessment of study quality using a prevalence quality assessment tool (PDF 52 kb)

Additional file 2: **Table S2.** Search Strategies. Search terms used for electronic databases Pubmed and Scopus (PDF 73 kb)

## Abbreviations

Anti-HCV: hepatitis C virus antibody
Anti-HDV: anti-hepatitis C virus antibody
ART: antiretroviral therapy
DNA: deoxyribonucleic acid
EIA: Enzyme immunoassay
HBsAg: hepatitis B surface antigen
HBV: hepatitis B virus
HCC: hepatocellular carcinoma
HCV: hepatitis C virus
HDV: hepatitis D virus
KCH: Kamuzu Central Hospital, Lilongwe, Malawi (National tertiary referral hospital)
MoH: Ministry of Health
PCR: polymerase chain reaction
QECH: Queen Elizabeth Central Hospital, Blantyre, Malawi (National tertiary referral hospital)
RNA: ribonucleic acid
WHO: World Health Organisation
