# Supplementary material for: Epidemiology of hepatitis B, C and D in Malawi: systematic review
Source: BMC Infect Dis. 2018 Oct 12;18:516. doi: 10.1186/s12879-018-3428-7 (PMC6186098; doi:10.1186/s12879-018-3428-7)
Supplement: Supplementary file 1 — Table S1. Assessment of quality of included studies, Assessment of study quality using a prevalence quality assessment tool (PDF 52 kb) [file 12879_2018_3428_MOESM1_ESM.pdf]

SUPPLEMENTARY TABLE 1: SEARCH STRATEGIES

Limits: Publication date: 1 January 1990 to 22 June 2018

No language restriction

**PUBMED** (https://www.ncbi.nlm.nih.gov/pubmed/)
((Malawi AND (hepatitis B[MeSH] or hepatitis C[MeSH] or hepatitis D[MeSH] or Hepatitis, Viral, Human[MeSH] or hepatitis or HBV or HBsAg or or HCV or anti-HCV or HCV antibody or core HCV antigen or HCVcAg or HCV RNA or HDV or anti-HD or anti-HDV or HDV IgG or viral hepatitis)))
**SCOPUS** (https://www.scopus.com/)
TITLE-ABS-KEY(malawi) AND (TITLE-ABS-KEY (hepatitis B) OR TITLE-ABS-KEY (hepatitis C) OR TITLE-ABS-KEY (hepatitis D) or TITLE-ABS-KEY (Hepatitis) OR TITLE-ABS-KEY (HBV) or TITLE-ABS-KEY (HBsAg) or TITLE-ABS-KEY (HCV) or TITLE-ABS-KEY (anti-HCV) or TITLE-ABS-KEY (HCV antibody) or TITLE-ABS-KEY (core HCV antigen) or TITLE-ABS-KEY (HCVcAg) or TITLE-ABS-KEY (HCV RNA) or TITLE-ABS-KEY (HDV) or TITLE-ABS-KEY (anti-HD) or TITLE-ABS-KEY (anti-HDV) or TITLE-ABS-KEY (HDV IgG))
