# Supplementary material for: Epidemiology of hepatitis B, C and D in Malawi: systematic review
Source: BMC Infect Dis. 2018 Oct 12;18:516. doi: 10.1186/s12879-018-3428-7 (PMC6186098; doi:10.1186/s12879-018-3428-7)
Supplement: Supplementary file 2 — Table S2. Search Strategies. Search terms used for electronic databases Pubmed and Scopus (PDF 73 kb) [file 12879_2018_3428_MOESM2_ESM.pdf]

SUPPLEMENTARY TABLE 2: Assessment of quality of included studies

	Was the sample representative of the target population	Were study participants recruited in an appropriate way?	Was the sample size adequate?	Were the study subjects and setting described in detail?	Was the data analysis conducted with sufficient coverage of the identified sample?	Were valid methods used for the identification of the condition?	Was the condition measured in a standard, reliable way for all participants?	Was the response rate adequate, and if not, was the low response rate managed appropriately?
Ahmed, 1998	Y	Y	Y	Y	Y	Y	Y	Y
Andreotti, 2014	Y	Y	Y	Y	Y	Y	Y	U
Aoudjane, 2014	Y	Y	Y	Y	Y	Y	Y	U
Candotti, 2001	N	U	N	N	U	Y	Y	U
Chasela, 2014	Y	Y	Y	Y	Y	Y	Y	Y
Chimphambano, 2007	N	Y	Y	Y	U	Y	Y	U
Chipetah, 2017	N	Y	Y	Y	Y	Y	Y	Y
Demir, 2016	Y	Y	Y	Y	Y	Y	Y	Y
Fox, 2015	N	Y	Y	Y	U	Y	Y	U
Greer, 2017	Y	Y	Y	Y	Y	Y	Y	Y
Loarec 2017	Y	U	Y	Y	U	Y	Y	U
Maida, 2000	N	Y	N	Y	U	Y	Y	U
Moore, 2010	U	Y	Y	Y	Y	Y	Y	Y
Nyirenda, 2008	N	Y	N	Y	U	Y	Y	U
Stockdale, 2017	Y	Y	Y	N	Y	Y	Y	U
Sutcliffe, 2002	N	U	Y	Y	U	Y	Y	U
Taha, 2015	Y	Y	N	N	U	Y	Y	U
Varo, 2016	Y	Y	U	Y	U	Y	Y	U

Abbreviations: Y Yes; N No; U Unclear
